# Evaluation of the protective effect of melatonin on oocyte, embryo and ovarian tissue parameters in female mice exposed to acetamiprid

**DOI:** 10.5935/1518-0557.20220068

**Published:** 2023

**Authors:** R Hassanzadeh, GA Joursaraei, LB Hejazian, F Feazi, H Najafzadehvarzi

**Affiliations:** 1 Student of Department of Anatomical Sciences, School of Medicine, Babol University of Medical Sciences, Babol, Iran; 2 Professor of Anatomical Sciences Department of Anatomical Sciences, School of Medicine, Infertility and Reproductive Health Research Center Health Research Institute, Babol University of Medical Sciences, Babol, Iran; 3 Assistant Professor of Anatomical Sciences Department of Anatomical Sciences, School of Medicine, Cellular and Molecular Biology Research Center Health Research Institute, Babol University of Medical Sciences, Babol, Iran; 4 Assistant professor of Medical Histology Department of Anatomical Sciences ‘School of Medicine Cellular and Molecular Biology Research Center Health Research institute, Babol University of Medical Science, Babol, Iran; 5 Cellular and Molecular Biology Research Center, Health Research Institute, Babol University of Medical Sciences, Babol, Iran

**Keywords:** melatonin, acetamiprid, ovary, IVF, mice

## Abstract

**Objective:**

Acetamiprid (ACP) causes infertility and its effect appears to occur via oxidative stress. Melatonin has antioxidative properties. Thus, in this experimental study, we examined the protective effect of melatonin against toxic pathologic changes from ACP on reproductive system parameters of female mice.

**Methods:**

The study included 30 female mice divided into 5 groups (6 mice in each group), as follows: Saline (control group); ACP (10, 20 mg/kg); ACP (10mg/kg) + melatonin (10mg/kg); and ACP (20mg/kg) + melatonin (10mg/kg). All mice were given intraperitoneal injections daily for one month. The groups were evaluated for ovarian histopathological changes and oocyte quality based on in vitro fertilization (IVF) parameters.

**Results:**

ACP induced histological damages in the ovaries of mice and caused increases in the number of atretic follicles and decreases in the quality of oocytes based on IVF parameters. These alterations were significantly reduced by melatonin.

**Conclusions:**

Melatonin can decrease the toxic effects of ACP in the female reproductive system of mice. Further studies are needed.

## INTRODUCTION

Infertility is described as the inability to achieve pregnancy after 12 months of regular sex without using contraceptive methods. Approximately 8-12% of couples of childbearing age are infertile (Mnif *et al*., 2011; Vander Borght & Wyns, 2018). Pesticides used in agriculture may cause infertility. Today, many pesticides are used to control pests and increase agricultural production. Different studies have linked pesticides to male infertility, miscarriage, and fetal defects (Yarinia *et al*., 2017; Vander Borght & Wyns, 2018).

Neonicotinoids are a class of pesticides that act as agonists of nicotinic acetylcholine receptors. They are widely used for the protection of crops and domestic purposes (Vander Borght & Wyns, 2018; Karaca *et al*., 2019). Acetamiprid (ACP) or (E) N1 [(6chloro3pyridyl) methyl]-N2-cyano-N1-methylacetamidine is a neonicotinoid. It is commonly used to protect leafy and fruiting vegetables, cabbages, citrus fruits, soft seeded fruits, grapes, cotton, ornamental plants, and flowers against sucking-type insects and fleas. ACP is water soluble and easily absorbed orally. Some studies showed that ACP can accumulate in human tissues (Karaca *et al*., 2019) and cause toxic effects on several organ systems such as the nervous, respiratory, immune, and reproductive systems (Arıcan *et al*., 2020). Adverse effects of ACP on human sperm, mouse oocyte, and reproductive function have also been reported (Abou Zeid, 2017). Other authors have associated ACP with embryonic abnormalities, including absence of some of the breast ribs or rib adhesions, soft and arcuate ribs, protrusion of the vertebrae, and lack of ossification of the palatal cartilage to the periosteum (Kimura-Kuroda *et al*., 2012; Gawade *et al*., 2013). ACP induces oxidative stress by reducing superoxide dismutase (SOD) and hepatic catalase activity while concomitantly increasing lipid peroxidation (Abou Zeid, 2017). Many scavenging agents and antagonists have been introduced to reduce pesticides toxicity (Ibrahim *et al*., 2012). Melatonin, a product of the pineal gland, participates in many important physiological functions. This neurohormone is a powerful natural antioxidant that not only has a direct antioxidant effect (due to reaction with ROS) (Vander Borght & Wyns, 2018; do Nascimento Marinho *et al*., 2019), but also decreases free radical levels by stimulating antioxidative enzymes activity (Nie *et al*., 2018). Melatonin may improve mitochondrial oxidative stress in the ovaries and protect oocytes against oxidative stress, especially during ovulation, and reduce symptoms of ovarian aging, decrease the number of follicles, decrease ovary size, decrease the number of blastocysts, and reduce mitochondrial function of these cells. In addition, melatonin has a protective effect on the fetus (Tan *et al*., 2003; Reiter *et al*., 2014; Zhang *et al*., 2019).

This study was designed to evaluate the protective effects of melatonin on oocyte, embryo and ovarian tissue parameters in female mice exposed to ACP. In vitro fertilization (IVF) was performed to evaluate the quality of the oocytes of the included mice (Van Voorhis, 2007).

## MATERIALS AND METHODS

The Institutional Research Ethics Committee approved this study and granted it certificate no. IR.MUBABOL.HRI.REC.1398.224. The study included 30 adult NMRI female mice weighing 30.5 g. The mice were divided into 5 groups (6 in each group). The first group received normal saline (solvent). Two other groups received acetamiprid (ACP) at doses of 10 mg/kg and 20 mg/kg (Singh *et al*., 2012). Another group was treated with 10 mg/kg of melatonin (Kurcer *et al*., 2010) and 10 mg/kg of ACP. And the mice in the last group received 10 mg/kg of melatonin and 20 mg/kg of ACP. All mice were given intraperitoneal injections daily for one month.

### Sperm preparation

The adult male mice were euthanized according to ethical principles. The skin of the scrotum was cut in half to show the testicles and the epididymis. The epididymis and vas deferens were also removed and placed in culture medium. After washing, the tissues were fragmented and placed in a Falcon tube with culture medium. The specimens were incubated at 37°C for 30 minutes. During incubation, sperm cells swam up from the tissue and floated in culture medium. In the end, the supernatant was collected.

### Oocyte preparation

Ovarian stimulation was performed at the end of the treatment period with intraperitoneal injections of human menopausal gonadotropins (HMG) at a dose of 7.5 IU. The mice were in ovulation stimulation for 48 hours. After 12 to 16 hours, human chorionic gonadotropin (HCG) was injected (15 units in each mouse). Then, the oocytes were placed inside the oviduct. The mice were euthanized according to ethical principles and the ovary and oviduct tissue were removed. The oviduct was separated from the ovary and placed inside Ham’s F10 culture medium. After washing with culture medium, the oocytes were removed from the oviduct under a loop microscope. The oocytes were washed into droplets of culture medium and divided into groups.

### IVF processing

The oocytes obtained in each group were randomly placed in five cross-sections of culture medium. Sperm cells were then drawn with a pasteurized pipette and injected in small amounts into each cross-section, until a cloud-like halo appeared around the oocytes. Then the culture was incubated at 37°C and 5% CO_2_. Culture incubation continued until cell division. Fertilization rate and cell division up to the blastocyst stage were assessed and formed embryos were categorized.

### Histological study

The ovarian tissues of the included mice were removed and placed in 10% formalin solution. After fixing, processing, and paraffin-embedding, 5-µm thick serial sections were obtained and the prepared sections were stained using hematoxylin-eosin and Mason trichrome.

### Analysis assay

The images of the stained slides were recorded with a camera and the number of primary, secondary, and antral follicles were evaluated using software package Image J. Software package Graph Pad Prism 6 was used in statistical analysis and graphing. In the tables and graphs, all the results are expressed as means ± standard deviation. One-way ANOVA and Tukey’s post-hoc test were used to compare the means between different groups. In all studies, *p*≤0.05 was considered statistically significant.

## RESULTS

### Ovarian histopathology

**Figure 1:**
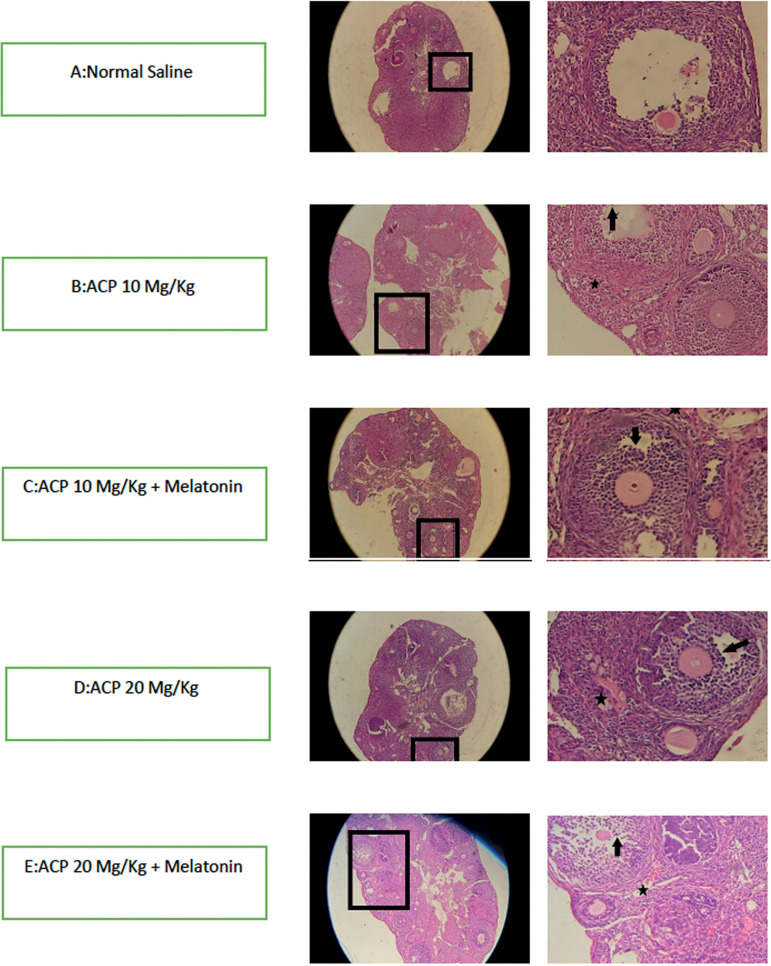
Histopathology examination of ovarian tissue by H&E staining. Inflammation and hyperemia are seen in all groups, except for the group given saline. Vascular hyperemia and inflammation are more severe in the groups given high-dose ACP. * indicates hyperemia and ↓ indicates inflammatory cells.

**Figure 2:**
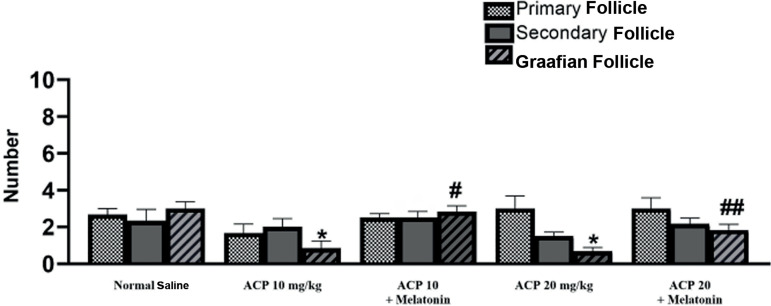
Mean number of primary, secondary and Graafian follicles. Data are in the form of means ± standard deviation. (*)indicates a significant difference in relation to the group given saline. (#)indicates a significant difference in relation to the group given ACP 10mg/kg. (##)indicates a significant difference in relation to the group given ACP 20mg/kg. (*p* ≤ 0.05).

Histological examination of the ovarian tissue of mice in the control group revealed a normal oval shape of the ovaries, a normally shaped tunica albuginea with a white appearance, and a normal stroma with numerous follicles containing female gametes in various stages of development in the ovarian cortex (Figure 1A). Histopathology analysis of ovarian tissue sections of the mice exposed to ACP showed abnormalities as compared to controls. Inflammation was the most common change seen in the ovarian tissue of mice from all groups. Inflammatory cells were seen in both ovarian stroma and follicular fluid in the follicles. Androgen-secreting cells and vascular hyperemia were present in the medulla area of the ovaries and peripheral area of the follicles (Figures 1B and D). Greater degrees of inflammation and vascular hyperemia were seen in mice exposed to high levels of ACP compared to mice given melatonin (Figures 1C and E). Follicle count was significantly greater in follicles in all stages in the two groups that received melatonin compared to mice given ACP. The number of Graafian follicles in the groups given low and high doses of ACP was significantly lower compared to mice given saline; however, such reduction was not statistically significant compared to mice given melatonin as a protective compound (Figure 2).

### IVF results

**Figure 3:**
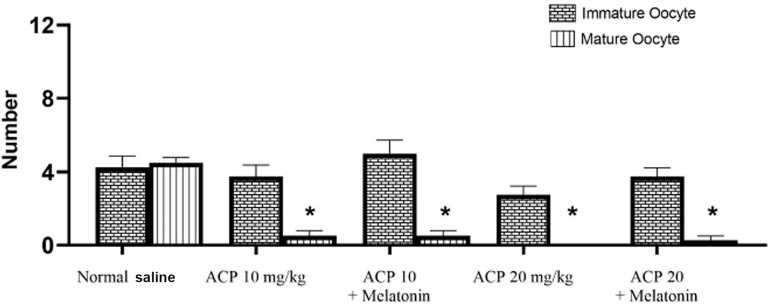
Mean number of immature and mature oocytes. Data are in the form of means ± standard deviation. (*)indicates a significant difference in relation to the group given saline. (*p*≤0.05).

**Figure 4:**
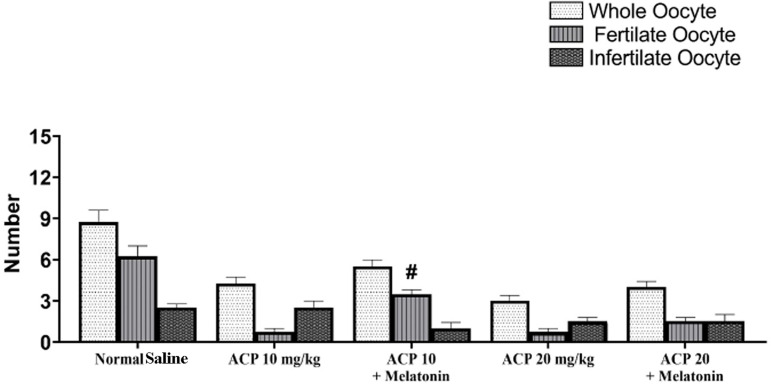
Mean number of total oocytes and number of fertile and infertile oocytes. Data are in the form of means ± standard deviation. (#)indicates a significant difference in relation to the gorup given saline. (*p*≤0.05).

After counting adult (M II) oocytes and immature oocytes (M I, GV), we found that the number of mature oocytes was significantly lower than in the group given saline (*p*≤0.05). The number of mature oocytes in the mice given ACP in both doses was significantly lower than in mice given saline (*p*=0.001). There was a slight decrease in the number of mature oocytes compared to the normal saline group, but this difference was not statistically significant (*p*>0.05) (Figure 3). In counting the total number of oocytes, the number of fertilized oocytes was considered and the difference between the two was the number of infertile oocytes. Oocyte counting was performed in all groups on the first, second, and third day of the study. The total number of oocytes in the groups given ACP was statistically lower than the number observed in the group given saline. The number of oocytes was significantly lower in the group given a low dose of ACP with melatonin than in the group given ACP alone at a dose of 10 mg/kg. This condition was significantly observed in the comparison between the group given melatonin with a high dose of ACP and the group given only a high dose of ACP. The mean number of fertilized oocytes was significantly lower when the low-dose and high-dose ACP groups were compared to the group given saline. However, the difference seen in the groups that received melatonin and ACP together was not statistically significant. The difference in the number of infertile or unfertilized oocytes was not significant between groups (*p*>0.05) (Figure 4).

**Figure 5:**
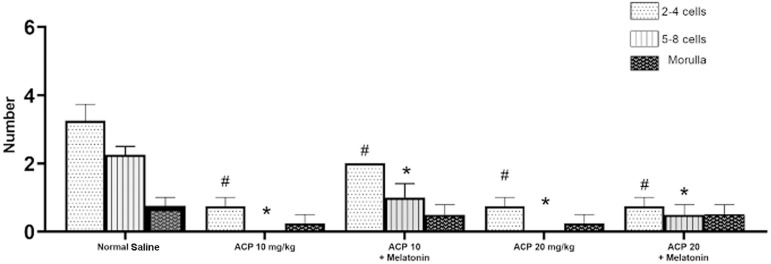
Comparison of IVF results. Data are in the form of means ± standard deviation. (*) & (#) indicate a significant difference in relation to the group given saline. (*p*≤0.05).

The number of blastomeres was counted after IVF. The embryos were divided into three groups based on the number of blastomeres: 2 to 4 cells; 5 to 8 cells; and morula-blastocysts. The number of 2-to-4-cell embryos in the groups given a low dose of ACP with melatonin and the group given saline was not statistically different. However, the number of 2-to-4-cell embryos in the groups given low or high doses of ACP and high doses of ACP with melatonin was significantly lower than the number seen in the group given saline (*p*≤0.05). The number of 5-to-8-cell embryos in the groups given low-dose ACP with melatonin was not statistically significant compared to the group given saline. However, the decrease in the number of 5-to-8-cell embryos in the high and low dose ACP groups and the high dose ACP with melatonin group was statistically significant (*p*-0.07). The number of embryos that reached the morula or blastocyst stage was not significantly different between the groups compared to the group given saline (*p*>0.05) (Figure 5).

## DISCUSSION

Pesticides are one of the most widely used chemicals that humans are directly or indirectly exposed to. Remnants of these compounds are found in water, soil, plants, and even animals. The adverse effects of these compounds on animals and humans are among the reasons why researchers pay attention to pesticides. In the first part of the present study, the damage caused by acetamiprid (ACP) on the ovarian and uterine tissue of female mice was investigated. ACP is a pesticide of the neonicotinoid family. Recent research has described the persistent presence of traces of these compounds in various tissues, including the liver, kidneys, and thyroid gland, to name a few (Arıcan *et al*., 2020).

In this study, ACP was administered to mice intraperitoneally at doses of 10 and 20 mg/kg. In ovarian tissue studies, hyperemia and inflammation were significantly observed. Also, decreases in the number of secondary follicles and Graafian follicles were observed. Due to these changes, it can be said that ACP damages follicles by disrupting the proliferation of follicular cells. These changes decrease the number of normal Graafian follicles and may increase the number of degenerated follicles. The decrease seen in the number of oocytes released after two doses of ACP reinforces the hypothesis that ACP inhibited ovulation. Lack of ovulation by ACP exposure can occur due to follicular damage and abnormal Graafian follicles. ACP may also disrupt follicular growth. Another possibility is that the oocytes may have degenerated before being released and making their way into the oviduct. The number of oocytes fertilized by IVF that developed into 4-to-8 cell embryos was lower in the group receiving 20 mg of ACP than in the group given saline, an indication of decreased oocyte quality.

Other studies have shown that ACP and other nicotine-like pesticides may produce adverse effects on mammalian reproductive organs, such as delayed testicular growth, impaired spermatogenesis, poor sperm quality, and changes in ovarian morphology (Bal *et al*., 2012). Another study showed that ACP has harmful effects on the mammalian reproductive system and affects the process of ovulation and the production of hormones that affect ovulation (Devan *et al*., 2015). Studies have suggested that the main mechanisms for ACP-induced toxicity include increased oxidative stress, decreased activity of antioxidant systems that play a protective role in the body (ROS), and increased lipid peroxidation of membranes. Ishikawa *et al*. (2015) evaluated the effect of ACP on the maturation of porcine oocytes in a laboratory. The results showed that oocyte nucleus maturation in the group given ACP at a concentration of 10 ppm was not significantly different from controls. However, concentrations of 30 ppm and 100 ppm significantly reduced oocyte maturation. Although the exact mode of action of ACP on oocyte maturation is unknown, occurrence of increased lipid peroxidation and decreased antioxidant factors including glutathione and super oxidase, catalase, glutathione peroxidase has been described (Ishikawa *et al*., 2015).

Based on these results, in the next step of this study, the effect of melatonin on the injuries caused by ACP was investigated. Melatonin is a natural hormone that is secreted by the pineal gland and some tissues of the body such as the eyes, intestines, and kidneys. On account of its anti-apoptotic and antioxidant properties, melatonin has been used to treat a variety of conditions for about a decade (Mnif *et al*., 2011). To investigate the protective role of melatonin against ACP toxicity, a dose of 10 mg of melatonin along with two doses of 10 and 20 mg of ACP/ kg were injected into mice daily. The results showed a positive effect of melatonin in reducing the severity of ACP-induced damage, with less severe epithelial destruction, inflammation, and hyperemia. However, the degree to which melatonin can compensate for the damages caused by a dose of 20 mg of ACP was lesser. In groups that received melatonin, the number of primary, secondary, and Graafian follicles increased compared to groups receiving only ACP. It seems that in counting the number of follicles, we found a protective effect for melatonin.

The results of these studies show that, despite the observation of some damage, melatonin ultimately had a positive and thought-provoking effect on the maintenance of the structure of follicles and oocytes. In examining the previous parameters, since only existing follicles were examined, statistical tests showed some negative effects and drawbacks of melatonin. Since the degenerated follicles that were lost had to be examined, a false negative effect may have been introduced. However, when the number of follicles was considered, we found that ACP significantly degenerated many follicles. This is why they were not included in the count. But melatonin, as a protective agent, prevented the degeneration of more follicles alone, especially when taken with ACP.

Melatonin belongs to the set of antioxidant enzymes including superoxidase, glutathione peroxidase, glutathione reductase and catalase. Melatonin has been shown to protect the structure of the ovaries and reduce oxidative stress (Ma *et al*., 2014). Melatonin has been shown to improve semen quality in vitro by neutralizing reactive oxygen species and nitrogen species in animal and human studies. Studies in rats have also shown that melatonin has a positive effect on improving sperm count, viability, and motility of sperm exposed to oxidative stress (Cheuquemán *et al*., 2015). Another study found that melatonin was associated with decreased levels of an oxidizing agent called oHdG-8 in oocytes (Karihtala *et al*., 2011). And as an antioxidant in the ovarian follicles, it helps in the maturation of ovulation, fetal growth and luteinization of granulosa cells. The results of a study by Ghasemi *et al.* described the protective effect of melatonin on nicotine-induced toxicity and showed that melatonin caused a significant normalization in the number of ovarian follicles and estradiol levels in group receiving nicotine and melatonin together. Our findings are consistent with this study, and in both groups in which melatonin was administered we found a significant improvement in the number of oocytes and follicles (Mohammadghasemi *et al*., 2012). Long Gino *et al*. mentioned that melatonin inhibits mutations during oocyte development (Jin *et al*., 2020).

The last step in our study was to investigate the effects of melatonin on IVF success. The division of the obtained embryos into groups with 2-4 cells, 5-8 cells, and embryos in the morula and blastocyst stage did not follow a specific article or reference. Rather, we found from experience that within the first two days of fertilization embryos are usually in the 2-4 cell stage, in a process that ultimately led to the other stages on the third and fourth days and subsequently from the fifth day onwards. Therefore, based on this method, embryos were included in different groups. The number of 2-to-4 cell embryos in the groups receiving low or high doses of ACP and a high dose of ACP with melatonin was statistically lower than in the saline group. This finding indicates that many fertilized oocytes did not seem to be able to reach more advanced division stages. If this hypothesis is correct, it indicates the effect of various toxins, including ACP, which either affected fertilization or stopped the formation of 2PN (diploid) embryos at the same stage. The groups given melatonin performed similarly to the group given saline. On the other hand, the groups given melatonin and low doses of ACP maintained a number of 2-to-4 cell embryos comparable to the counts seen in the saline group. The non-significant difference observed between these groups can be, in some way, credited to melatonin. The number of 2-to-4 cell embryos was significantly greater than in the group treated ACP alone, indicating a positive effect of melatonin on IVF outcome. Mature oocytes and improved oocyte quality increase the success rates of assisted reproductive technology (ART) treatments (Sun *et al*., 2020).

## CONCLUSION

Melatonin played a protective role against female reproductive system toxicity by ACP exposure. However, it should be noted that melatonin administration was ineffective in some parameters and did not differ significantly from the group receiving the toxin. More studies are needed to confirm our findings.

## References

[r1] Abou Zeid SM (2017). Developmental Toxicity of Acetamiprid in Rats. World J Pharm Pharm Sci.

[r2] Arıcan EY, Gökçeoğlu Kayalı D, Ulus Karaca B, Boran T, Öztürk N, Okyar A, Ercan F, Özhan G. (2020). Reproductive effects of subchronic exposure to acetamiprid in
male rats. Sci Rep.

[r3] Bal R, Naziroğlu M, Türk G, Yilmaz Ö, Kuloğlu T, Etem E, Baydas G. (2012). Insecticide imidacloprid induces morphological and DNA damage
through oxidative toxicity on the reproductive organs of developing male
rats. Cell Biochem Funct.

[r4] Cheuquemán C, Arias ME, Risopatrón J, Felmer R, Álvarez J, Mogas T, Sánchez R. (2015). Supplementation of IVF medium with melatonin: effect on sperm
functionality and in vitro produced bovine embryos. Andrologia.

[r5] Devan RKS, Mishra A, Prabu PC, Mandal TK, Panchapakesan S. (2015). Chemistry. Sub-chronic oral toxicity of acetamiprid in Wistar
rats. Toxicol Environ Chem.

[r6] do Nascimento Marinho KS, Lapa Neto CJC, de Sousa Coelho IDD, da Silva MA, Gomes Melo ME, Dos Santos KRP, Chagas CA, Coelho Teixeira ÁA, Teixeira VW. (2019). Genotoxic and mutagenic evaluation of the protective effect of
exogenous melatonin in adult rats and their offspring exposed to the
insecticides methomyl and cypermethrin during pregnancy. Mutat Res Genet Toxicol Environ Mutagen.

[r7] Gawade L, Dadarkar SS, Husain R, Gatne M. (2013). A detailed study of developmental immunotoxicity of imidacloprid
in Wistar rats. Food Chem Toxicol.

[r8] Ibrahim NA, GabAllah AM, Hendawy HS. (2012). Humoral immune response to acetamiprid exposure and its
modulation by supplementation with vitamin C in rats. Biochem Lett.

[r9] Ishikawa S, Hiraga K, Hiradate Y, Tanemura K. (2015). The effects analysis of two neonicotinoid insecticides on in
vitro maturation of porcine oocytes using hanging drop monoculture
method. J Vet Med Sci.

[r10] Jin L, Zhu HY, Kang XJ, Lin LP, Zhang PY, Tan T, Yu Y, Fan Y. (2020). Melatonin protects against oxybenzone-induced deterioration of
mouse oocytes during maturation. Aging (Albany NY).

[r11] Karaca BU, Arican YE, Boran T, Binay S, Okyar A, Kaptan E, Özhan G. (2019). Toxic effects of subchronic oral acetamiprid exposure in
rats. Toxicol Ind Health.

[r12] Karihtala P, Kauppila S, Puistola U, Jukkola-Vuorinen A. (2011). Divergent behaviour of oxidative stress markers
8-hydroxydeoxyguanosine (8-OHdG) and 4-hydroxy-2-nonenal (HNE) in breast
carcinogenesis. Histopathology.

[r13] Kimura-Kuroda J, Komuta Y, Kuroda Y, Hayashi M, Kawano H. (2012). Nicotine-like effects of the neonicotinoid insecticides
acetamiprid and imidacloprid on cerebellar neurons from neonatal
rats. PLoS One.

[r14] Kurcer Z, Hekimoglu A, Aral F, Baba F, Sahna E. (2010). Effect of melatonin on epididymal sperm quality after testicular
ischemia/reperfusion in rats. Fertil Steril.

[r15] Ma M, Chen XY, Gu C, Xiao XR, Guo T, Li B. (2014). Biochemical changes of oxidative stress in premature ovarian
insufficiency induced by tripterygium glycosides. Int J Clin Exp Pathol.

[r16] Mnif W, Hassine AI, Bouaziz A, Bartegi A, Thomas O, Roig B. (2011). Effect of endocrine disruptor pesticides: a
review. Int J Environ Res Public Health.

[r17] Mohammadghasemi F, Jahromi SK, Hajizadeh H, Homafar MA, Saadat N. (2012). The Protective Effects of Exogenous Melatonin on Nicotine-induced
Changes in Mouse Ovarian Follicles. J Reprod Infertil.

[r18] Nie J, Xiao P, Wang X, Yang X, Xu H, Lu K, Lu S, Liang X. (2018). Melatonin prevents deterioration in quality by preserving
epigenetic modifications of porcine oocytes after prolonged
culture. Aging (Albany NY).

[r19] Reiter RJ, Tamura H, Tan DX, Xu XY. (2014). Melatonin and the circadian system: contributions to successful
female reproduction. Fertil Steril.

[r20] Singh TB, Mukhopadhayay SK, Sar TK, Ganguly S. (2012). Acetamiprid induces toxicity in mice under experimental
conditions with prominent effect on the hematobiochemical
parameters. J Drug Metab Toxicol.

[r21] Sun TC, Liu XC, Yang SH, Song LL, Zhou SJ, Deng SL, Tian L, Cheng LY. (2020). Melatonin Inhibits Oxidative Stress and Apoptosis in
Cryopreserved Ovarian Tissues via Nrf2/HO-1 Signaling
Pathway. Front Mol Biosci.

[r22] Tan DX, Manchester LC, Hardeland R, Lopez-Burillo S, Mayo JC, Sainz RM, Reiter RJ. (2003). Melatonin: a hormone, a tissue factor, an autocoid, a paracoid,
and an antioxidant vitamin. J Pineal Res.

[r23] Van Voorhis BJ (2007). Clinical practice. In vitro fertilization. N Engl J Med.

[r24] Vander Borght M, Wyns C. (2018). Fertility and infertility: Definition and
epidemiology. Clin Biochem.

[r25] Yarinia M, Amirahmadi M, Ostadgholami M, Babaei M, Emami A, Elmi M, Shoeibi S. (2017). Simultaneous Analysis of Seven Non-authorized Pesticides Residue
in Cucumber Using Spiked Calibration Curve by GC/ECD. Iran J Toxicol.

[r26] Zhang L, Zhang Z, Wang J, Lv D, Zhu T, Wang F, Tian X, Yao Y, Ji P, Liu G. (2019). Melatonin regulates the activities of ovary and delays the
fertility decline in female animals via MT1/AMPK pathway. J Pineal Res.

